# Inter-relationships between Gender, Frailty and 10-Year Survival in Older Italian Adults: an observational longitudinal study

**DOI:** 10.1038/s41598-019-54897-2

**Published:** 2019-12-05

**Authors:** Graziamaria Corbi, Francesco Cacciatore, Klara Komici, Giuseppe Rengo, Dino Franco Vitale, Giuseppe Furgi, Gennaro Pagano, Leonardo Bencivenga, Sergio Davinelli, Nicola Ferrara

**Affiliations:** 1 Dept of Medicine and Health Sciences, University of Molise, and Italian Society of Gerontology and Geriatrics (SIGG), Campobasso, Italy; 2 Dept of Translational Medical Sciences, Federico II University of Naples, Naples, Italy; 3 Istituti Clinici Scientifici Maugeri SpA Società Benefit” (ICS Maugeri SpA SB), Telese Terme, (BN) Italy; 4 Casa di Cura San Michele, Maddaloni, (CE) Italy; 5 Dept of Basic & Clinical Neuroscience Institute of Psychiatry, Psychology & Neuroscience (IoPPN) King’s College, London, UK

**Keywords:** Geriatrics, Disease-free survival, Epidemiology

## Abstract

Aim of the present study was to assess the impact of gender on the relationship between long-term mortality and clinical frailty. In an observational, longitudinal study on 10-year mortality, we examined 1284 subjects. The Frailty Staging System was used to assess frailty. The Cox model was employed to assess variables independently associated with survival using a backward stepwise algorithm. To investigate the possible interactions between gender and the selected variables, an extension of the multivariable fractional polynomial algorithm was adopted. Women were more likely to be older, have a higher disability, present with more comorbidities, consume more drugs, be frail and have a higher rate of survival at the follow-up than were men. At the Cox multivariate analysis only age (HR 2.26), female gender (HR 0.43), and number of drugs (HR 1.57) were significant and independent factors associated with all-cause mortality. In the survival analyses, only frailty (*vs* no frailty) showed significant interaction with gender (p < 0.001, HR = 1.92). While the presence of frailty reduced the survival rate in women, no effect was observed in men. Importantly, frail women showed higher survival rates than did both frail and no frail men. The main finding of the present study is that gender shapes up the association between frailty and long-term survival rates.

## Introduction

Over the last few decades, the growing burden of ageing has caused a dramatic change in demographic features. Globally, the older population increased from 9% in 1994 to 12% in 2014, with a projection of over 80 years reaching 19% in 2050^1^. The older population is predominantly composed of females worldwide; 54% of the individuals aged 60 and over are females, and 62% of the individuals over 80 years old are females. These percentages are destined to increase in the coming decades^1^. The reduced mortality observed over time in older individuals suggests that with appropriate interventions, it is possible to substantially increase longevity^2^. However, due to the high prevalence of chronic non-communicable diseases, especially in the elderly population, it is not fully clear if the increase in life expectancy is associated with increases in years in a healthy status and quality of life. While health declines with ageing, this decline is even more significant in the oldest population, which is characterized by a health status that is worse in women than in men and in individuals with a low level of education than in those with a high level of education^3^.

However, there are several differences in risk factors, the amount of environmental exposure, and levels of vulnerability between women and men that are often poorly recognized. Indeed, despite their longer life expectancy than men, women have less access to health care^4^, have higher levels and a higher percentage of body fat^5^ and present lower physical activity levels than men of all ages, suggesting the relevance of biological adaptations that might explain their advantage in terms of lifespan^6^.

Physical and cognitive impairments^7^, as well as frailty^8^, induce disability and reduce quality of life differently in men than in women. The Women’s Health Initiative Observational Study enrolled 40,000 women aged 65–70 years; 16.3% of the participants were classified as frail, 28.3% were considered pre-frail upon enrolment in the study, and incident frailty was identified in 14.8% of the participants at the 3-year follow-up. The participants who developed frailty were older and less educated than the other participants, were smokers and were treated with hormone replacement therapy^9^.

However, the impact of gender on the relationship between long-term mortality and clinical frailty has not yet been investigated. Indeed, while several studies have investigated the differences in survival between frail and non-frail older adults, there is a lack of data on the impact of gender on this relationship. Only one recent study, by Zhang *et al*.^10^, has used gender as a discriminating variable to investigate gender-related factors associated with frailty and their impact on mortality. However, no studies are available on the influence of gender on changes in the long-term survival of elderly individuals with or without frailty.

Thus, the aim of the present study was to assess the impact of gender on the relationship between long-term mortality and clinical frailty.

## Results

The survival status, which was evaluated until the end of 2003 by means of death certificates, was obtained for 1293 out of the 1332 (97.1%) subjects that were initially enrolled. Of these 1293 individuals, data on social support were unavailable in 9 subjects (0.06%). Thus, our final study population consisted of 1284 subjects (552 men and 732 women), with a mean age of 74.19 ± 6.38 years. Over a mean follow up of 101.9 ± 44.9 months (range: 6–146 months), 680 (53.0%) deaths were observed (323 [47.5%] in men and 357 [52.5%] (p < 0.001) in women, with a final cumulative mortality rate of 56.3 ± 1.5% (95% CI 56.22 56.38) and a median survival time of 126 months. Table 1 depicts the main baseline characteristics of the study population. The women were more likely to be older, have a higher BMI, have higher rates of hypertension, have diabetes and neurological diseases, have lower rates of COPD, use more drugs and present a higher prevalence of frailty than were men (Table 1). Nevertheless, the women exhibited a higher 10-year survival than the men (105.59 ± 44.75 *vs* 97.09 ± 44.66 months; p = 0.001).

**Table 1:** Main characteristics of 1,284 older subjects by gender

	All (n = 1,284)	Men (n = 552)	Women (n = 732)	p
Age, years mean (SD)	74.19 (6.38)	73.70 (5.97)	74.55 (6.65)	**0.018**
BMI, kg/m2 mean (SD)	26.56(4.92)	25.89(4.06)	27.07(5.42)	**<0.001**
Waist circumference, cm mean (SD)	95.75(16.28)	96.27(15.47)	95.35(16.89)	0.358
Charlson comorbidity index	1.64 (1.67)	1.56 (1.56)	1.70 (1.75)	0.115
Drugs number, mean (SD)	2.28(2.06)	2.01(1.94)	2.48(2.14)	**<0.001**
Hypertension, n (%)	970 (75.5)	393 (71.2)	577 (78.8)	**0.002**
Diabetes, n (%)	187 (14.6)	62 (11.2)	125 (17.1)	**0.003**
CAD, n (%)	70 (5.5)	46 (8.3)	24 (3.3)	**<0.001**
CHF, n (%)	120 (9.3)	48 (8.8)	72 (10.0)	0.447
COPD	488 (38.0)	268 (48.6)	220 (30.1)	**<0.001**
CKD, n (%)	53 (4.1)	25 (4.5)	28 (3.8)	0.530
Neurological diseases, n (%)	168 (13.1)	56 (10.1)	112 (15.3)	**0.007**
Frail, % yes (n)	531 (41.4)	163 (29.5)	368 (50.3)	**<0.001**
Survival, months mean (SD)	101.94 (44.89)	97.09 (44.66)	105.59 (44.75)	**0.001**

Abbreviations: BMI, Body Mass, Index; CAD, Coronary Artery Disease; CHF, Chronic Heart Failure; COPD, Chronic Obstructive Pulmonary Disease; CKD, Chronic Kidney Disease. In bold the significant p values are reported.

As shown in Supplementary Table 1, which reports a comparison between survivors and non-survivors stratified by gender, as expected, the women were more likely to be alive at the 10-year follow-up than were the men; however, surprisingly, the women that were alive showed worse clinical conditions than the men did, as indicated by the higher prevalence of comorbidities, including diabetes and neurological diseases, the higher prevalence of frailty and the more frequent use of medications (Supplementary Table 1).

**Figure 1:**
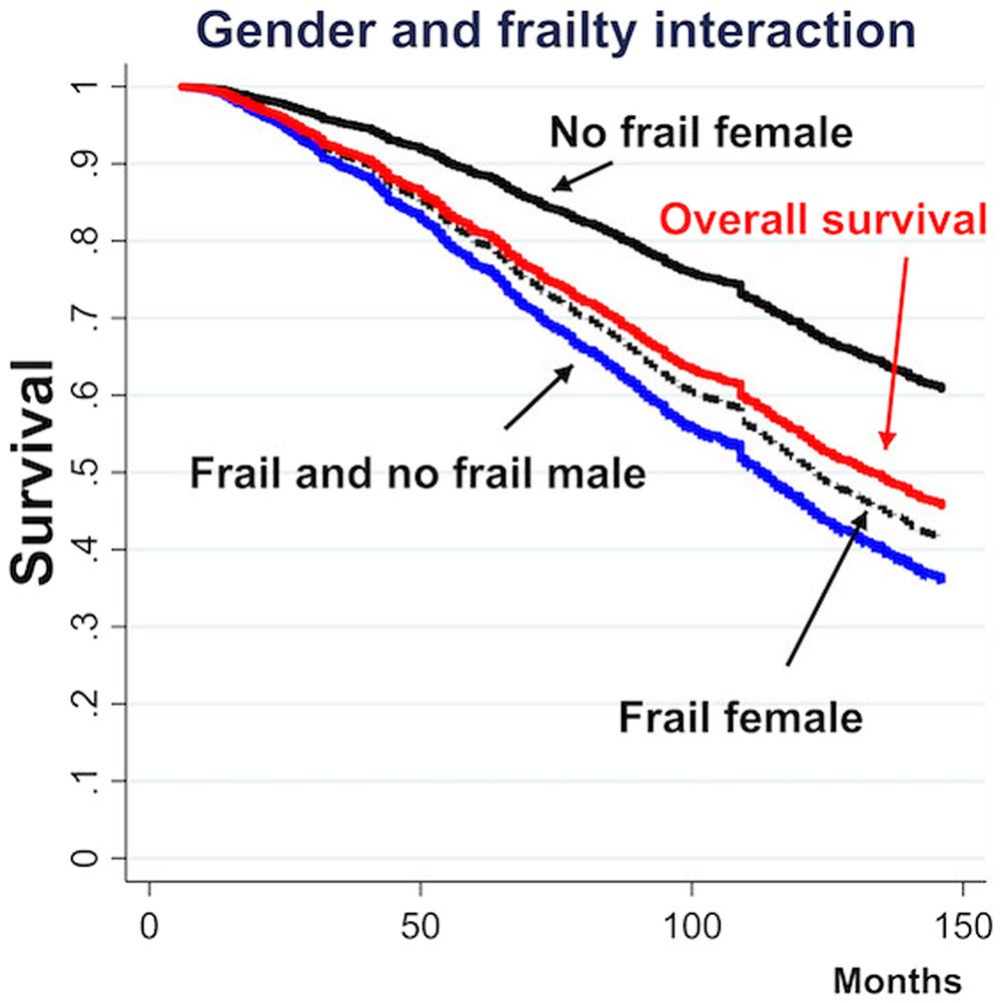
Gender and frailty interaction directly adjusted survival curves. Each directly adjusted curve, estimated at a specific factor and interaction value, is compared to the overall observed survival Kaplan Meier curve (red continuous line) and exemplifies the survival that would be observed if all patients in the study population had had the given specific factor and interaction value. Continuous black line = no frail female. Dashed black line = frail female. Continuous blue line = no frail male. Dashed blue line = frail male. The male lines fully overlap.

Cox proportional analysis was adjusted for age, gender, BMI, waist circumference, comorbidities, number of drugs and frailty status. All of these parameters have been demonstrated to affect the health status of older subjects in the literature (Table 2). Among the thirteen factors included in the model, only age (HR 2.26; 95% CI 1.799–2.840), female gender (HR 0.43; 95% CI 0.299–0.561), and number of drugs (HR 1.57; 95% CI 1.093–2.047) were significant and independent factors associated with all-cause mortality. The multivariate model showed a global R^2^ of 29%, suggesting that a good fraction of the outcome variability was associated with mortality. Interestingly, the contributions of age, gender, number of drugs and frailty toward the variation of the prognostic index, as documented by the partial R^2^, were 46.6%, 14.6%, 25.8%, and 13.0%, respectively. Of note, only frailty showed a significant interaction with gender, exhibiting an HR of 1.92 (95% CI 1.56 2.37, p < 0.001). The visual impact of the frailty-gender interaction on survival is shown in Fig. 1, where the survival curves relative to the frailty and gender interaction are reported along with the overall Kaplan Maier curve. While frail women had a significantly worse survival rate than non-frail women did (p < 0.001, HR = 0.50), the presence of frailty did not impact mortality in men. Importantly, the survival rate of frail women was higher than that of men independent of frailty.

**Table 2:** Cox survival analysis

	HR	95% CI		p	% fraction of global R^2^	Bootstrap Inclusion Frequency (%)	Linearity Stability (%)	Interaction with gender	
		Lower	Higher					HR	p
Age (*referred to 10 years*)	2.26	1.799	2.840	**≤0.001**	46.6	100	82.0	0.994	0.559
Gender (*referred to male*)	0.43	0.299	0.561	≤0.001	14.6	99.9	NA	—	—
BMI (kg/m^2^)	0.90	0.755	1.073	0.24		56.4	NA	1.004	0.780
Waist circumference (cm)	1.1	0.666	1.818	0.71		41.2	NA	1.000	0.922
Drugs number	1.57	1.093	2.047	**≤0.001**	25.8	98.7	98.6	0.876	0.432
Hypertension (*referred to no Hypertension*)	0.85	0.701	1.032	0.10		8.7	NA	0.916	0.606
Diabetes *(referred to no Diabetes*)	1.3	0.989	1.709	0.06		55.7	NA	0.791	0.125
CAD (*referred to no CAD*)	1.1	0.709	1.705	0.67		14.4	NA	1.68	0.111
CHF (*referred to no CHF*)	1.25	0.982	1.592	0.07		69.0	NA	0.761	0.144
COPD (*referred to no COPD*)	1.1	0.942	1.285	0.23		10.5	NA	0.804	0.055
CKD (*referred to no CKD*)	0.9	0.607	1.334	0.6		12.7	NA	1.191	0.533
Neurol. disease (*referred to no Neurol disease*)	1.1	0.904	1.338	0.34		22.2	NA	0.891	0.462
Frailty (*referred to no Frailty*)	1.02	0.771	1.351	0.89	13.0	91.2	NA	1.92	**≤0.001**

HR, Hazard Ratio; 95% CI, 95% Coefficient Interval; R^2^ = explained variance. BMI, Body Mass Index; CAD, Coronary Artery Disease; CHF, Chronic Heart Failure; COPD, Chronic Obstructive Pulmonary Disease; CKD, Chronic Kidney Disease; Neurol. disease, Neurological disease. NS = Not Significant. NA = Not Applicable.

## Discussion

In the present study, we have shown that the female gender is associated with a longer 10-year survival rate than is the male gender in older adults. Moreover, the presence of frailty had a negative impact on the survival rate in women, and as depicted by the survival curves (Fig. 1), mortality rates were similar in frail women and men (frail and not frail). Thus, frailty could minimize the advantage of the female gender over the male gender in terms of survival. Indeed, it is important to emphasize that frailty does not affect mortality in men.

Our results are partially consistent with those of a recent study by Zhang *et al*.^10^ on 1953 subjects enrolled in the National Health and Nutrition Examination Survey (NHANES) programme. In particular, the authors demonstrated that the prevalence of frailty increased with age in both males and females but was higher in females than in males. The presence of frailty had a negative impact on survival in both men and women, whereas mortality rates were higher in men than in women. Furthermore, in our study, we found that frailty was more prevalent in women than in men, but mortality was higher in men than in women. However, Zhang *et al*.^10^ enrolled a population with a lower prevalence of frailty (7% of the 1953 participants were considered frail) than that of the present investigation (41% of the 1284 participants were considered frail). Moreover, the follow-up period of 3 years is another relevant difference between the study by Zhang *et al*. and our investigation.

The different impacts of frailty on survival in males between the study by Zhang *et al*. and our study may be explained by the differences in performance of some frailty scores used to identify the presence of this condition in men. Recently, Pijpers *et al*.^11^ demonstrated that frailty instruments are not sufficiently sensitive for screening and diagnostic purposes, suggesting that these tools can be used to exclude rather than to identify frailty^11^. Moreover, Dent *et al*.^12^ proposed that a specific frailty instrument can be effective to a certain extent in recognizing frailty in different clinical settings and/or in specific subpopulations, suggesting that some frailty scores can be used for screening and others can be used for a full assessment^12^. One possible explanation for the influence of gender on long-term survival rates, which was observed in our study, is that the majority of frailty indexes might be more prone to detect frailty in women than in men.

Other possible explanations are related to the well-recognized concept that frailty involves a complex interplay among several factors (physiological, medical, environmental and social) responsible for an altered capacity to respond to stress. Indeed, different definitions of frailty share the same idea that frailty is a multifactorial phenomenon^13,14^. Moreover, frailty is a dynamic process involving changes over time; thus, longitudinal assessments of the different components are often necessary^15^. Of note, frailty prevalence varies extensively based on the assessment instrument employed^16^. For instance, several studies have compared the “phenotype” and “deficit model” approaches in identifying older individuals at a high risk of death^17–20^, and despite the evident correlation between the approaches, the “deficit model” approach better predicts mortality than the “phenotype” approach^17,18^. Theou *et al*. suggested that the great majority of studies employing different criteria for the definition of frailty resulted in substantial differences in prevalence, the influence of gender, and the ability to predict mortality^21^. In our study, a key element influencing the 10-year survival rate is the female gender. In this regard, it is important to emphasize that systematic reviews have shown that the prevalence of frailty is higher in women than in men, independent of the instrument of frailty adopted^22,23^. The FSS, which was used in this study, has a different capacity to identify the presence of frailty in men than in women, and this difference in capacity is probably related to gender-related factors implicated in the genesis of frailty. In fact, the ageing process is also characterized by important biological and behavioural differences linked to gender that might affect the incidence, pathophysiology and progression of several widespread diseases^24,25^. García-González *et al*.^26^ described that frailty is higher in women than in men. The authors have speculated that women accumulate more deficits at a faster speed in late life than do men^26^; this difference can be explained by the higher rate of disability in women than in men^27^, which is possibly related to genetic^28^ and social factors^26,29^. In the data of 1413 older adults, Alexandre *et al*.^30^ searched for gender-related differences in the incidence of frailty and for the main determinants of the frailty phenotype (low physical activity, unintentional weight loss, slowness, exhaustion and weakness). Unintentional weight loss and slowness were the main determinants of frailty in men, while all components of frailty except for weakness had relevance in women^30^. Recently, Zhang *et al*.^10^ found that different factors were implicated in the onset of frailty; marital status, daily total calorie intake, and smoking were the most important factors in men. In contrast, obesity, a high CRP, a family history of diabetes and a family history of a heart attack were the most important factors in women^10^. The last study is the only study that was made available before our study and used gender as a discriminating variable to assess survival, but as mentioned above, it included only a few frail subjects and a shorter follow-up period than our study did. A recent meta-analysis confirmed that women tolerate frailty better than men do; this finding was demonstrated by a lower mortality rate in women than in men at any level of frailty or age, which suggests differences linked to gender are involved in the well-known male-female health-survival paradox^31^. Similarly, we found that women are more likely to be older, have a higher prevalence of frailty and comorbidities, and be treated with more drugs than are men; these findings are in line with the well-recognized scenario that the older population is mainly composed of females and individuals who are older, have disabilities, have a large number of comorbidities and are undergoing poly-pharmacotherapy.

### Limitations and strengths

The main limitation of the present study is the unavailability of other frailty indexes to evaluate the impact of gender on 10-year survival rates. Although we have reported results from an observational study, another limitation may be the retrospective nature of our investigation. However, it is important to underline the absence of studies that investigated survival rates after 10 years in a population of elderly subjects and those that specifically tested the influence of gender and frailty on prognostic risk stratification.

### Conclusions

This is the first study showing that gender shapes up the association between frailty and long-term survival rates. However, additional long-term perspective studies are needed to confirm the role of gender in long-term survival rates and to better identify the gender-related components that affect frailty. In fact, the identification of these elements alone may help to better prevent frailty and adequately manage the older frail population.

## Methods

### Ethical approval and informed consent

The “Osservatorio Geriatrico Regione Campania” study received full ethical approval from the Research Ethics Committee of the University of Naples Federico II, which approved patients’ information and consent forms. All study procedures were conducted in accordance with the ethical standards of the committee responsible for human experimentation (institutional and national) and with the Helsinki Declaration of 1975, which was revised in 2013. Informed consent was obtained from all patients included in the study. This report adheres to the consolidated standards for the reporting of longitudinal studies and was written according to the STROBE guidelines for Observational Studies in Epidemiology^32^ (see the checklist in the Supplemental file).

### Study Population

The “Osservatorio Geriatrico Regione Campania” was a longitudinal study that evaluated 10-year mortality rates and was designed to evaluate the prevalence of chronic conditions, impairments, disabilities, and drug consumption in a region of South Italy (Campania). The study design has been previously described^33^. In summary, the study sample consisted of 1780 subjects aged 65 and older who were randomly selected from the electoral rolls and were living within the five provinces of Campania; the subjects were stratified by a three-step procedure according to age, sex, and size of the urban unit. Of the 1780 patients, 448 (25.2%) refused to participate; the final study sample included 1332 subjects, representing an overall participation rate of 74.8%.

### Data Collection

At baseline, physicians were trained to administer a questionnaire, which included cognitive and depression tests and questions regarding social status and demographic variables (sex, age, marital and educational status), and they examined the subjects. Chronic conditions were evaluated from the medical histories and confirmed by clinical examinations by a physician. The diagnoses of myocardial infarction and peripheral artery disease were made by the Rose questionnaire^34^ and a clinical examination. Drugs taken by the subjects were also reported. Comorbidities were evaluated using the Charlson Comorbidity Index^35^. The Italian version of the Mini-Mental State Examination (MMSE) validated by Measso *et al*^36^. was used as a measure of cognitive mental status; cognitive impairment was defined by a score <24 according to Folstein^37^. Subjects were screened for depression using the Geriatric Depression Scale (GDS) reported by Yesavage^38^. A GDS score between 0 and 10 was defined as normal, a score between 11 and 20 was indicative of moderate depression, and a score >20 was indicative of severe depression. Disability was evaluated using both the Activity of Daily Living (ADL) and Instrumental Activity of Daily Living (IADL) tools^39^. Subjects who could not perform the activities without assistance were considered disabled.

Social support was assessed using a validated questionnaire^40^, which evaluated social networks, social relationships, and economic support. The total score ranged from 0 to 17, in which the lowest social support level corresponded to the highest score. The score was divided into tertiles: 0–5 corresponded to the highest amount of social support (score = 1), 6–12 corresponded to a medium amount of social support (score = 2) and 13–17 corresponded to the lowest amount of social support (score = 3)^41^.

### Frailty staging system

The “clinical” term frailty has been defined by the Frailty Staging System (FSS)^42^, which combines seven functional domains: disability, mobility, cognitive function, visual function (scored from 1 = no visual impairment to 4 = blindness), hearing function (scored from 1 = no hearing problem to 4 = total deafness), urinary continence and social support (scored from 4 = the highest support to 1 = the lowest support)^41^. On the basis of previous work^33^, the present investigation considered frail subjects who needed assistance as patients with at least one BADL, poor mobility (assessed by evaluating the ability to perform heavy housework, to walk up and down stairs to the second floor and to walk half a mile; score 3 or 4), visual impairment (score 3 or 4), cognitive impairment (MMSE score <24), hearing impairment (score 3 or 4), urinary incontinence (total incontinence) and low social support (score 4)^33^. Subjects who were frail in one or more of these domains were classified as frail, in accordance with a previous study^33,41,43^.

### Statistical analysis

Continuous variables are expressed as the mean ± standard deviation (SD), while categorical variables are expressed as proportions. The Cox proportional hazard model was adopted to assess the independent association of each factor with the survival probability and to select, from 13 candidates (the variables shown in Table 1 including gender), the significant variables with a backward stepwise algorithm. Then, we adopted the multivariable fractional polynomial (MFP) algorithm^44^, which further allows assessment of the functional form (linearity/nonlinearity) of each continuous factor. To investigate the possible interactions between gender and the variables selected by the MFP algorithm, an extension of the MFP was adopted (MFPI)^45^. The model building procedure included the following steps: 1. Assessment of the discrimination ability of the final Cox model by a measure of the explained variance of the model (R^2^) as proposed by Royston and Sauerbrei^46^. The goodness of fit was assessed by the Gronnesby and Borgan calibration test; 2. Evaluation of the weight of each significant factor included in the final model by means of the hazard ratio and by the amount of global variance explained (R^2^). The assessment of the global R^2^ was accomplished by the Shapley-Owen decomposition algorithm^47^; 3. Verification of the proportionality assumption of the Cox model using a modified version of the MFP (the MFPT) that allows both the assumption to be verified and an extended Cox model to be built if necessary; 4. Assessment of the internal validity of the final model by evaluating the stability of its characteristics with nonparametric bootstrap sampling^48^. Briefly, given the parameters of the model obtained with the aforementioned model-building procedure, the stability of each factor tested in the model (including the interaction terms) was measured by the frequency that each factor was selected as significant in a large (1000) number of bootstrap replications of the dataset (BIF).

To compare estimates at specific factor values of the survival curve adjusted for the other significant factors of the final model, plots obtained with the directly adjusted method were adopted^49^. Data were analysed by STATA version 15.0 (StataCorp LP, College Station, Texas). Statistical significance was accepted at p < 0.05. Patients with missing data from the follow-up or baseline assessments were excluded from the statistical analysis.

## Supplementary information

Supplemental file

## Data Availability

The datasets generated during and/or analysed during the current study are available from the corresponding author on reasonable request.
