# Supplementary material for: Inter-relationships between Gender, Frailty and 10-Year Survival in Older Italian Adults: an observational longitudinal study
Source: Sci Rep. 2019 Dec 5;9:18416. doi: 10.1038/s41598-019-54897-2 (PMC6895198; doi:10.1038/s41598-019-54897-2)
Supplement: Supplementary file 1 — Supplemental file [file 41598_2019_54897_MOESM1_ESM.pdf]

Inter-relationships between Gender, Frailty and 10-Year Survival in Older Italian Adults: an observational longitudinal study

Graziamaria Corbi, Francesco Cacciatore, Klara Komici, Giuseppe Rengo, Dino Franco Vitale, Giuseppe Furgi, Gennaro Pagano, Leonardo Bencivenga, Sergio Davinelli, Nicola Ferrara

Supplemental Table 1. Characteristics of study population at 10-year survival stratified by health status and gender

**Variables**	**Alive**				**Dead**			
	**All** *(n=604)*	**Men** *(n=229)*	**Women** *(n=375)*	***p***	**All** *(n=680)*	**Men** *(n=323)*	**Women** *(n=357)*	***p***
**Age, *years* mean (SD)**	71.65 (4.89)*	71.32(4.47)*	71.86(5.12)*	.190	76.44(6.69)	75.39(6.31)	77.39(6.89)	**<.001**
**BMI, *kg/m²* mean (SD)**	26.73 (4.70)	26.09(4.17)	27.11 (4.96)	**.014**	26.41 (5.09)	25.75 (3.99)	27.03 (5.88)	**.001**
**Waist circumference, *cm* mean (SD)**	95.40 (15.87)	96.45(15.14)	94.77 (16.29)	.247	96.06 (16.65)	96.15 (15.73)	95.98 1(7.51)	.903
**Charlson comorbidity index, mean (SD)**	1.27 (1.42)*	1.10 (1.11)*	1.38 (1.57)*	**.018**	1.98 (1.81)	1.88 (1.74)	2.05 (1.87)	.228
**Drugs number, mean (SD)**	1.87 (1.81)*	1.58 (1.69)*	2.04(1.86)*	**.002**	2.65 (2.20)	2.32 (2.04)	2.95 (2.31)	**<.001**
**Hypertension, n (%)**	450 (74.5)	165 (72.1)	285 (76.0)^	.280	520 (76.5)	228 (70.6)	292 (81.8)	**.001**
**Diabetes, n (%)**	62 (10.3)*	14 (6.1)*	48 (12.8)*	**.009**	125 (18.4)	48 (14.9)	77 (21.6)	**.024**
**CAD, n (%)**	23 (3.8)*	11 (4.8)*	12 (3.2)	.318	47 (6.9)	35 (10.8)	12 (3.4)	**<.001**
**CHF, n (%)**	27 (4.5)*	6 (2.6)*	21 (5.7)*	.080	93 (13.9)	42 (13.2)	51 (14.7)	.579
**COPD, n (%)**	191 (31.6)*	97 (42.2)	94 (25.1)	**<.001**	297 (43.7)	171 (52.9)	126 (35.3)	**<.001**
**CKD, n (%)**	20 (3.3)	9 (3.9)	11 (2.9)	.507	33 (4.9)	16 (5.0)	17 (4.8)	.908
**Neurological diseases, n (%)**	66 (10.9)*	17 (7.4)	49 (13.1)	**.031**	102 (15.0)	39 (12.1)	63 (17.6)	**.042**
**Frail, *n (%)***	163 (27.0)*	36 (15.1)*	127 (33.9)*	**<.001**	368 (54.1)	127 (39.3)	241 (67.5)	**<.001**

BMI, Body Mass, Index; CAD, Coronary Artery Disease; CHF, Chronic Heart Failure; COPD, Chronic Obstructive Pulmonary Diseases; CKD, Chronic Kidney Disease

* p <0.05 versus dead; ^ p=0.05

Inter-relationships between Gender, Frailty and 10-Year Survival in Older Italian Adults: an observational longitudinal study

Graziamaria Corbi, Francesco Cacciatore, Klara Komici, Giuseppe Rengo, Dino Franco Vitale, Giuseppe Furgi, Gennaro Pagano, Leonardo Bencivenga, Sergio Davinelli, Nicola Ferrara.

STROBE Statement

Checklist of items that should be included in reports of observational studies

Section/Topic	Item No	Recommendation	Reported on Page No
**Title and abstract**	1	(*a*) Indicate the study's design with a commonly used term in the title or the abstract	1
		(*b*) Provide in the abstract an informative and balanced summary of what was done and what was found	2
**Introduction**			
Background/rationale	2	Explain the scientific background and rationale for the investigation being reported	3,4
Objectives	3	State specific objectives, including any prespecified hypotheses	4
**Methods**			
Study design	4	Present key elements of study design early in the paper	10
Setting	5	Describe the setting, locations, and relevant dates, including periods of recruitment, exposure, follow-up, and data collection	10-14
Participants	6	(*a*) *Cohort study*—Give the eligibility criteria, and the sources and methods of selection of participants. Describe methods of follow-up	10-14
		(*b*) *Cohort study*—For matched studies, give matching criteria and number of exposed and unexposed	NA
Variables	7	Clearly define all outcomes, exposures, predictors, potential confounders, and effect modifiers. Give diagnostic criteria, if applicable	10-14
Data sources/measurement	8*	For each variable of interest, give sources of data and details of methods of assessment (measurement). Describe comparability of assessment methods if there is more than one group	11,12
Bias	9	Describe any efforts to address potential sources of bias	NA
Study size	10	Explain how the study size was arrived at	11,12
Quantitative variables	11	Explain how quantitative variables were handled in the analyses. If applicable, describe which groupings were chosen and why	10-14
Statistical methods	12	(*a*) Describe all statistical methods, including those used to control for confounding	12-14
		(*b*) Describe any methods used to examine subgroups and interactions	12-14
		(*c*) Explain how missing data were addressed	12-14
		(*d*) *Cohort study*—If applicable, explain how loss to follow-up was addressed<br>*Case-control study*—If applicable, explain how matching of cases and controls was addressed<br>*Cross-sectional study*—If applicable, describe analytical methods taking account of sampling strategy	NA
		(*e*) Describe any sensitivity analyses	NA
